# Virus induced gene silencing (VIGS) for functional analysis of wheat genes involved in *Zymoseptoria tritici* susceptibility and resistance

**DOI:** 10.1016/j.fgb.2015.04.006

**Published:** 2015-06

**Authors:** Wing-Sham Lee, Jason J. Rudd, Kostya Kanyuka

**Affiliations:** Plant Biology and Crop Science Department, Rothamsted Research, Harpenden AL5 2JQ, United Kingdom

**Keywords:** RNAi, *Mycosphaerella graminicola*, *Septoria tritici*, Wheat, *Triticum aestivum*, Virus induced gene silencing (VIGS), Functional genomics

## Abstract

Virus-induced gene silencing (VIGS) has emerged as a powerful reverse genetic technology in plants supplementary to stable transgenic RNAi and, in certain species, as a viable alternative approach for gene functional analysis. The RNA virus *Barley stripe mosaic virus* (BSMV) was developed as a VIGS vector in the early 2000s and since then it has been used to study the function of wheat genes.

Several variants of BSMV vectors are available, with some requiring *in vitro* transcription of infectious viral RNA, while others rely on *in planta* production of viral RNA from DNA-based vectors delivered to plant cells either by particle bombardment or *Agrobacterium tumefaciens*.

We adapted the latest generation of binary BSMV VIGS vectors for the identification and study of wheat genes of interest involved in interactions with *Zymoseptoria tritici* and here present detailed and the most up-to-date protocols.

## Highlights

• Virus-induced gene silencing (VIGS) is a powerful tool for studying gene function.
• *Barley stripe mosaic virus* (BSMV) vector is used for VIGS in wheat.
• We adapted BSMV VIGS to study wheat–*Zymoseptoria tritici* interactions.
• Here we present our detailed and the most up-to-date protocols for BSMV VIGS.

## 1 Introduction

*Zymoseptoria tritici*, also known as *Septoria tritici* and *Mycosphaerella graminicola*, is a filamentous ascomycete fungus that causes Septoria tritici leaf blotch (STB) disease, one of the most important foliar diseases of wheat worldwide (Dean et al., 2012). Infection biology of this fungus is relatively well studied and analysis of recently produced big ‘omics’ datasets, from approaches such as RNA sequencing, and comprehensive proteome and/or metabolome profiling are beginning to provide a wealth of candidate genes and signaling pathways with possible roles in STB disease establishment in wheat (Rudd et al., 2015; Yang et al., 2013).

STB disease control currently relies primarily on intensive fungicide use. However, the increasingly common reports of *Z. tritici* field isolates that have evolved fungicide resistance (Cools and Fraaije, 2008; Fraaije et al., 2005) and new European legislation due to come into force in the near future restricting the use of several commonly used fungicides (Hillocks, 2012), will almost certainly result in a shift towards more focus on breeding for STB disease resistance. At least fifteen different major resistance genes as well as a number of quantitative trait loci for STB resistance have already been identified (Chartrain et al., 2009; Ghaffary et al., 2012; Goodwin, 2007), but none of these have so far been cloned.

The ordered draft genome sequence of the hexaploid wheat (*Triticum aestivum*) reference genotype Chinese Spring has recently been produced by the International Wheat Genome Sequencing Consortium (IWGSC). Survey of the gene content and composition of all 21 wheat chromosomes identified and annotated 124,201 gene loci (IWGSC, 2014). This will facilitate the analysis of both RNA sequencing and genome re-sequencing data and enable researchers to identify and isolate their candidate genes of interest. One of the major challenges now for researchers is therefore to validate and functionally analyze the contribution of both candidate wheat disease resistance genes and candidate genes with predicted roles in wheat–*Z. tritici* interactions, with the aim of informing the development of new strategies for STB control. In model plant species and in crops with small genomes, functional analysis of candidate genes is often done through chemical-, T-DNA-, or transposon-aided mutagenesis, stable transgene overexpression, or stable RNA interference (RNAi). Some of these approaches are also available for wheat, but they remain expensive, time consuming and laborious. Virus-induced gene silencing (VIGS) has emerged in recent years as a relatively inexpensive and rapid tool for functional analyses of candidate genes in wheat (Lee et al., 2012). VIGS exploits a natural post-transcriptional gene silencing (PTGS) defense mechanism in plants that operates against virus infection (Waterhouse et al., 2001). All plant viruses produce either long double-stranded RNAs or highly structured single-stranded RNAs. These are detected by the plant host and trigger PTGS, which involves the generation of small interfering RNAs (siRNAs) that are then loaded into the RNA-induced silencing complex containing at least one Argonaute endonuclease to guide the cleavage of complementary viral RNA (Baulcombe, 2004). In VIGS, a fragment of a plant gene is inserted into a virus vector to form a recombinant virus that, upon infection of a plant host, induces PTGS targeting both the virus RNA and homologous endogenous plant RNA sequences for degradation (Lu et al., 2003). Only one plant virus, *Barley stripe mosaic virus* (BSMV), which has a tri-partite RNA genome comprising RNAα, RNAβ and RNAγ, has so far been developed into a VIGS vector for wheat (Scofield et al., 2005). BSMV-mediated VIGS has been successfully applied to improve our understanding of a number of wheat–pathogen interactions such as the wheat–leaf rust (Scofield et al., 2005), wheat–stripe rust (Wang et al., 2011) and wheat–powdery mildew (Geng et al., 2013) interactions, and to dissect a multitude of other traits unrelated to plant pathology (Lee et al., 2012).

Since the first report on the use of BSMV as a vector for VIGS in cereals (Holzberg et al., 2002) several improvements to the original vector have been carried out to improve the ease and costs of the VIGS procedure. One of the latest BSMV VIGS systems, developed by Yuan et al. (2011), allows *Agrobacterium tumefaciens* to be used to deliver binary BSMV VIGS vectors into host plant cells. We have adapted this system to enable functional analyses of candidate plant genes and dissection of signaling pathways involved in compatible or incompatible wheat–*Z. tritici* interactions.

Here, a detailed description of all steps required for targeted silencing of wheat leaf genes using this latest generation of BSMV VIGS vectors and for assessment of *Z. tritici* infection in conjunction with VIGS, is presented. A flow diagram of the steps described is presented in Supplemental Fig. 1.

## 2 Protocols

### 2.1 Step 1: Target sequence selection

Subject the whole coding sequence (CDS) of the target wheat gene of interest to an *in silico* analysis using si-Fi (siRNA Finder; http://labtools.ipk-gatersleben.de/) software to select 250–400 nt sequence regions that are predicted to produce high numbers of silencing-effective siRNAs. The likelihood of off-target silencing from different regions of the inputted sequence can also be evaluated using si-Fi. These analyses require a local sequence database in Fasta format representing all the mRNAs or gene CDS of the target organism to be loaded into si-Fi using the “Create new Database” option. In the absence of a complete wheat genome sequence one can currently use either the wheat UniGene set and/or the more comprehensive collection of confirmed and *in silico* predicted wheat gene CDS that are available for downloading from ftp://ftp.ncbi.nih.gov/repository/UniGene/Triticum_aestivum/ and ftp://ftp.ensemblgenomes.org/pub/plants/release-24/fasta/triticum_aestivum/cds/ (IWGSC, 2014), respectively. One can also evaluate the wheat gene of interest CDS for potential interspecific off-target silencing using either si-Fi or the Basic Local Alignment Search Tool (BLAST) analysis against the predicted *Z. tritici* transcriptome (Goodwin et al., 2011), which can be downloaded from http://fungi.ensembl.org/Zymoseptoria_tritici/Info/Index.

When possible, select at least two preferably non-overlapping regions of the gene of interest for VIGS analyses. Observation of the same phenotype induced by silencing using each of the two or more independent VIGS constructs is a good indication that the phenotype is due to specific silencing of the intended target gene, therefore allowing greater confidence in the obtained results.

When attempting to silence an individual member of a gene family consider selecting the sequences from the 3′- or 5′-UTR regions, which are generally more variable than the CDS. This should minimize the risk of off-target silencing. On the other hand, in cases when a great deal of functional redundancy is expected among different gene family members, it should be possible to design VIGS construct(s) from the conserved gene regions in order to target several or even all gene family members simultaneously.

Regarding VIGS experimental design, at least one negative control VIGS construct containing a 250–400 nt fragment of a non-plant origin gene, such as the *Aequorea victoria Green Fluorescent Protein* gene (*GFP*; GenBank accession E17099) or the *Escherichia coli β-glucuronidase* gene (*GUS*; GenBank accession AF305918) should be included. BSMV::*asTaPDS* or BSMV::*asTaChlH* constructs, which induce photobleaching or yellow-orange coloration of the silenced tissue (Lee et al., 2012) due to depletion of enzymes involved in biosynthesis of carotenoid pigments or chlorophyll, respectively, may be used as positive controls for gene silencing.

### 2.2 Step 2: VIGS constructs preparation

Target gene sequences for insertion into the BSMV VIGS vector are generally produced via RT-PCR amplification using total wheat RNA, extracted either from healthy leaves or from leaves infected with *Z. tritici*, as a template. The latter may be required when the target gene is known or suspected to be expressed at low levels in healthy tissue and up-regulated during *Z. tritici* infection. In cases when the selected sequence is located in a single exon, the sequence can also be obtained by PCR amplification from plant genomic DNA.

Clone the VIGS target sequences into the BSMV RNAγ vector pCa-γbLIC (Yuan et al., 2011) via ligation independent cloning (LIC), in either sense or antisense orientation. Antisense constructs may be slightly more efficient in inducing gene silencing (Steven R. Scofield, personal communication; Zhang et al., 2010). The following LIC adaptor sequences should be appended to the 5′-ends of the PCR primers used to amplify the VIGS target sequences: 5′-AAGGAAGTTTAA-3′ (forward primer) and 5′-AACCACCACCACCGT-3′ (reverse primer).

Resolve the PCR products by gel-electrophoresis in a 1.4% TBE agarose gel and purify using QIAquick Gel Extraction Kit (Qiagen) according to the manufacturer’s instructions. In parallel, linearize the pCa-γbLIC vector with *Apa*I (Sigma–Aldrich) at 25 °C for 2 h, before inactivating the enzyme by incubating at 65 °C for 20 min. Treat both the gel-purified PCR product and linearized pCa-γbLIC vector with T4 DNA polymerase to generate complimentary sticky ends. For this, incubate (1) ∼200–250 ng of the PCR product at 22 °C for 30 min in a total volume of 10 μl with 0.6 U of T4 DNA polymerase (New England Biolabs) in 1x reaction buffer supplemented with 100 ng/μl bovine serum albumin (BSA) and 5 mM dATP, and (2) 500 ng of *Apa*I-digested pCa-γbLIC at 22 °C for 30 min in a total volume of 50 μl with 3 U of T4 DNA polymerase in 1x reaction buffer supplemented with 100 ng/μl BSA and 5 mM dTTP. After incubation, heat-inactivate the T4 DNA polymerase at 75 °C for 15 min. Mix 10 μl treated PCR product (∼200–250 ng) and 2 μl of the treated pCa-γbLIC vector (20 ng) together, incubate at 65 °C for 2 min and then incubate for 10 min at room temperature to allow annealing of the complementary sticky ends. Any remaining treated pCa-γbLIC vector can be stored at −20 °C for future use. Use an 1.5–2 μl aliquot for transformation of 20 μl chemically competent *E. coli* JM109 (Promega). Plate the entire transformation mixture onto one or two LB agar plates supplemented with kanamycin (50 μg/ml), and incubate at 37 °C overnight.

Most of the resulting *E. coli* colonies should carry the desired recombinant pCa-γbLIC vector containing the target plant sequence insert. However, it is advisable to confirm transformants via colony PCR with primers 2235.F (5′-GATCAACTGCCAATCGTGAGTA-3′) and 2615.R (5′-CCAATTCAGGCATCGTTTTC-3′) that flank the LIC site in the pCa-γbLIC vector. Purify plasmid DNA from 2 to 3 independent, confirmed transformants using QIAprep Spin Miniprep Kit (Qiagen) as per product protocol and confirm the insert identity by sequencing the plasmid DNA with the 2235.F and/or 2615.R primer.

Transform the sequence verified pCa-γbLIC VIGS construct into *A. tumefaciens* GV3101 by electroporation. For this we use the MicroPulser (Bio-Rad) electroporator, 0.1 cm gap electroporation cuvettes, and home-made electro-competent cells produced according to the protocol described by Lin (1995) with the following modification: Agrobacterium cultures are grown to a final OD_600_ of 1.2 and the cells are pelleted by centrifugation and washed in ice-cold sterile 10% glycerol seven times in total. Electroporation is done using the manufacturer’s pre-set conditions for Agrobacterium i.e. one 2.2 kV pulse. Plate an aliquot of the transformation mixture on LB agar supplemented with 25 μg/ml gentamycin and 50 μg/ml kanamycin. As BSMV requires all three genomic segments, RNAα, RNAβ and RNAγ, for successful infection it is necessary to also produce *A. tumefaciens* GV3101 strains containing pCaBS-α (BSMV RNAα) and pCaBS-β (BSMV RNAβ).

### 2.3 Step 3: Preparation of virus inoculum and infecting wheat plants with BSMV

As wheat is recalcitrant to *A. tumefaciens* infection, BSMV is first introduced via agroinfiltration into the leaves of dicot host *Nicotiana benthamiana*. This plant species is fully susceptible to Agrobacterium. It is also able to support high levels of BSMV accumulation. Sap extracted from BSMV-infected *N. benthamiana* leaves is then used to rub-inoculate leaves of young wheat seedlings.

For *N. benthamiana* agroinfiltration, grow 5 ml cultures (LB supplemented with 25 μg/ml gentamycin and 50 μg/ml kanamycin) of *A. tumefaciens* strains carrying pCa-γbLIC VIGS constructs overnight at 28 °C with constant shaking at 220 rpm. For each BSMV RNAγ construct, BSMV RNAα and RNAβ constructs in 5 ml cultures will also be required. Pellet the *A. tumefaciens* cells at 2500 rcf for 20 min in a Centrifuge 5810 R (Eppendorf) or similar, re-suspend in infiltration buffer [10 mM MgCl_2_, 10 mM 2-(N-morpholino)ethanesulfonic acid (MES) pH 5.6, and 150 μM acetosyringone] to a final optical density at 600 nm (OD_600_) of 1.5, and incubate at room temperature without shaking for 3 h or longer.

Mix *A. tumefaciens* strains carrying BSMV RNAα, RNAβ, and RNAγ strains together in 1:1:1 ratio and pressure infiltrate the bacteria into the abaxial side of fully expanded leaves of approximately 25–30 days old *N. benthamiana* plants using a needleless 1-ml syringe. Use ∼0.5–1 ml of Agrobacterium suspension per leaf and aim to infiltrate the whole area of each leaf.

Harvest directly infiltrated *N. benthamiana* leaf tissue at 3–5 days post-agroinfiltration. This tissue can be used for preparing the virus inoculum straight away or snap frozen in liquid nitrogen and stored at -80 °C for later use. We suggest keeping the *N. benthamiana* plants after removal of the agroinfiltrated leaves for 2–4 more days. If the agroinfiltration was successful, BSMV-induced mosaic symptoms should become clearly visible in the upper uninoculated leaves by 7–9 days post-agroinfiltration.

Pre-germinate wheat seeds on pre-wetted filter paper or sterile sand. Select germinated seeds with root radicles at a similar point of emergence to equilibrate maturity of plants to be used in virus inoculations. Plant the germinated seeds in small seed trays (we use 22.5 cm × 17 cm × 6 cm trays) filled with plant growth media/compost suitable for cereals. Make a trench along the longer side of a tray at 1–2 cm from the side wall and plant 15–16 germinated seeds spreading them evenly along the trench. Prepare at least one tray for each experimental and each control VIGS construct. Cultivate wheat seedlings for 10–14 days or until the two leaf stage. Label each tray displaying the wheat genotype name and the VIGS construct the plants are to be inoculated with. Wheat genotypes we use routinely to study wheat–*Z. tritici* interactions are Bobwhite and Riband (cultivars susceptible to *Z. tritici* isolate IPO323), and Cadenza and Chinese Spring (cultivars resistant to *Z. tritici* isolate IPO323), but other wheat genotypes may also be used.

**Fig. 1:**
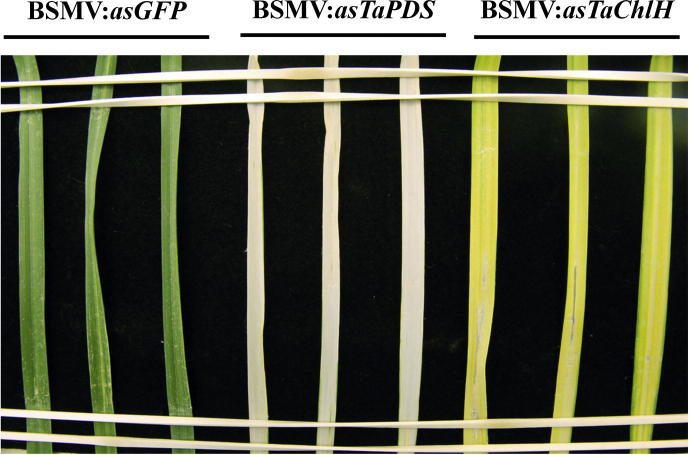
Wheat leaves pre-inoculated with various BSMV VIGS constructs aligned on a platform, ready for the *Z. tritici* challenge inoculation. Eleven days old seedlings of wheat (*Triticum aestivum*) were inoculated with the control virus construct BSMV:*asGFP*, or with BSMV:*asTaPDS* or BSMV:*asTaChlH*, which target the wheat *PDS* (*phytoene desaturase*) or *ChlH* (*magnesium chelatase subunit H*) genes involved in carotenoid and chlorophyll biosynthesis, respectively. Silencing of *PDS* results in photobleaching, whilst *ChlH*-silenced leaves turn yellow in color. Leaves shown are the third leaves of 25-days old plants (at 14 days post-virus inoculation) bent over a black platform ready for inoculation with *Z. tritici* conidiospore suspension. Three leaves per treatment are shown.

Grind virus-infected *N. benthamiana* leaf tissue using a pre-chilled mortar and pestle in 10 mM potassium phosphate buffer pH 7 containing 1–2% w/v Celite® 545 AW (Sigma–Aldrich) abrasive. Use 2.5–3 ml of buffer per 1 g of *N. benthamiana* leaf tissue. Inoculate the first and second leaves of young wheat plants with the sap as follows. Hold the lower stem of the plant firmly with one hand, dip forefinger of the other hand in the sap inoculum and give each leaf 3–6 relatively gentle passes between forefinger and thumb. A slight squeaking sound should be audible as the wax is being scraped off of the leaves. Let the plants absorb the virus for 5–10 min before spraying the leaves using a fine mist water spray bottle. Cover plants with plastic bags and allow to recover from the mechanical stress in low light (e.g. under the bench) overnight. You should see no or minimal damage to the leaves on the following day. Return plants to standard conditions favoring BSMV-mediated VIGS: photoperiod – 16 h, temperature – 20–23 °C (night-day), light intensity – 120–150 μmol m^–2^ s^−1^, and relative humidity – 50–60%. First symptoms of virus infection (yellow spots or stripes) should appear on upper uninoculated leaves by 5–11 days post inoculation, depending on the wheat genotype. Effects of silencing BSMV::*asTaPDS* and BSMV::*asTaChlH* should become apparent on the third and/or the fourth leaves by approximately 10–14 days post inoculation (Fig. 1).

### 2.4 Step 4: Assessment of virus-induced gene silencing

Successful silencing of the wheat gene targets in the VIGS construct-infected plants is assessed using quantitative reverse-transcription PCR (qRT-PCR). The primers used for this purpose should bind outside the region targeted for silencing. Samples for qRT-PCR analysis should ideally be taken from the same plant as that used in the *Z. tritici* bioassay, as the degree of gene silencing can vary between plants.

The usual guidelines for designing qRT-PCR assays for relative gene expression analyses apply (Bustin et al., 2009). The reference genes we use for target gene expression normalization in samples from *Z. tritici*-VIGS experiments are *TaCDC48* (UniGene Ta.46201) and *TaeIF4E* (UniGene Ta.48486) (Lee et al., 2014).

### 2.5 Step 5: Fungal inoculations and Septoria tritici blotch disease assessment

*Z. tritici* strain stocks are stored as conidiospore water suspensions in 50% (v/v) glycerol at −80 °C. For plant inoculation with the reference *Z. tritici* isolate IPO323, harvest conidiospores from 6 to 7 days old Yeast Peptone Dextrose (YPD) agar cultures grown at 15–17 °C into sterile water supplemented with 0.1% (v/v) Silwet L-77 (Lehle Seeds). Filter the suspension through one layer of Miracloth (Calbiochem) and adjust the fungal inoculum to 500,000 conidiospores per mL. This should be applied to the third or fourth leaves of virus-infected wheat plants at approximately 10–15 days post-VIGS construct inoculation.

**Fig. 2:**
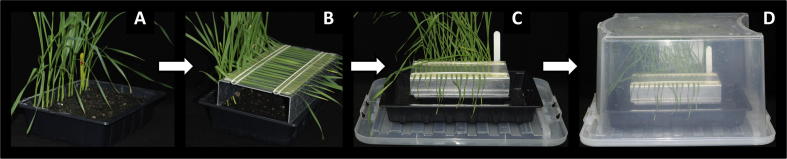
Experimental set-up for the STB disease attached wheat seedling leaf bioassay. (A) Wheat seedlings in small seed trays. (B) The third leaves of approximately 25-days old wheat seedlings bent over an inoculation platform and secured with rubber bands ready for inoculation with *Z. tritici* conidiospore suspension. (C) Immediately after inoculation with *Z. tritici*, the small seed tray of plants is placed into a larger seed tray which is then filled with water and placed on the lid of an incubation box. (D) *Z. tritici*-inoculated plants inside an incubation box.

Prepare the wheat plants for inoculation with *Z. tritici* by gently bending the third and/or fourth leaves of the wheat plants over a inoculation platform that is free-standing on the seed tray parallel to the row of growing wheat plants. Fasten the leaves adaxial side up, to the inoculation platform using double-sided sticky tape and rubber bands as shown in Fig. 2. Spread the fungal inoculum evenly onto the surface of the bent leaves using a cotton wool swab. Place the small seed tray into a larger seed tray (we use 36.5 cm × 22.5 cm × 5.5 cm trays) without drainage holes in the bottom, and fill the larger tray with water. Incubate the trays inside a suitably sized transparent plastic lidded box (Fig. 2) to retain high humidity for 72 h before returning the plants to standard growth conditions.

Visually monitor STB disease progression preferably on a daily basis, recording the first appearance of symptoms (commonly manifested as chlorotic flecks) and the subsequent development of irregular tan-colored necrotic lesions containing scatterings of black pycnidia (fungal fruiting bodies). Assess STB disease severity at 21–28 days post inoculation (if using *Z. tritici* IPO323) by scoring the percentage of leaf area covered by pycnidia-bearing lesions. In routine assessments we apply a step-wise scale between 0 (green leaf, no lesions) to 5 (80–100% of leaf area covered by pycnidia). Phenotyping can also be carried out using automated image analysis systems that allow more accurate quantification of pycnidia sizes and numbers (Stewart and McDonald, 2014).

Spore wash counts may be carried out to complement visual assessment of STB disease severity. Detach equally-sized *Z. tritici*-inoculated leaf segments (we use 6-cm long segments), each from an individual leaf, and place them in sterile tubes plugged with cotton wool saturated with sterile water. Replace the tube lids and incubate at 15–17 °C for 24–48 h in the dark. After this high relative humidity incubation discard the cotton wool plugs, add a (small) standard amount of water to each tube (in the region of 1–3 ml) and vortex vigorously. The pycnidiospores washed off the infected leaves are counted using a hemocytometer. Alternatively, the extent of plant colonization by *Z. tritici* can be assessed by quantifying the amount of fungal DNA in the infected leaf tissue using real-time quantitative PCR as described in Rudd et al. (2008).

## 3 Conclusions

Very little is known at the molecular level about how *Z. tritici* is able to evade recognition by host defenses during the early asymptomatic infection phase, and how it subsequently triggers host programmed cell death during the necrotrophic phase of infection. Agrobacterium-mediated transformation and/or random mutagenesis screens have enabled researchers to study the effect of disrupting gene function in the fungus on the outcome of *Z. tritici*–wheat interactions. However, the expense and difficulties of stable transformation in wheat has meant that elucidating the role of host plant genes in interactions with this fungus has been slow and laborious. We have adapted one of the new generation of binary BSMV VIGS systems for the study of wheat gene function in *Z. tritici*–wheat leaf interactions, allowing more rapid screening of larger numbers of candidate plant genes that are being identified from RNA sequencing, map-based cloning of disease resistance genes and resistance QTL analysis projects. We have described our routine protocols above for use by other researchers in the community, with the aim of increasing our collective understanding of plant genes involved in *Z. tritici*–wheat interactions and thereby informing the development of new strategies for STB control.
