# Supplementary material for: Virus induced gene silencing (VIGS) for functional analysis of wheat genes involved in Zymoseptoria tritici susceptibility and resistance
Source: Fungal Genet Biol. 2015 Jun;79:84–8. doi: 10.1016/j.fgb.2015.04.006 (PMC4510315; doi:10.1016/j.fgb.2015.04.006)

Supplemental Figure 1. A flow diagram of all the steps required for targeted silencing of wheat leaf genes using VIGS vectors and for assessment of *Z. tritici* infection in conjunction with VIGS.

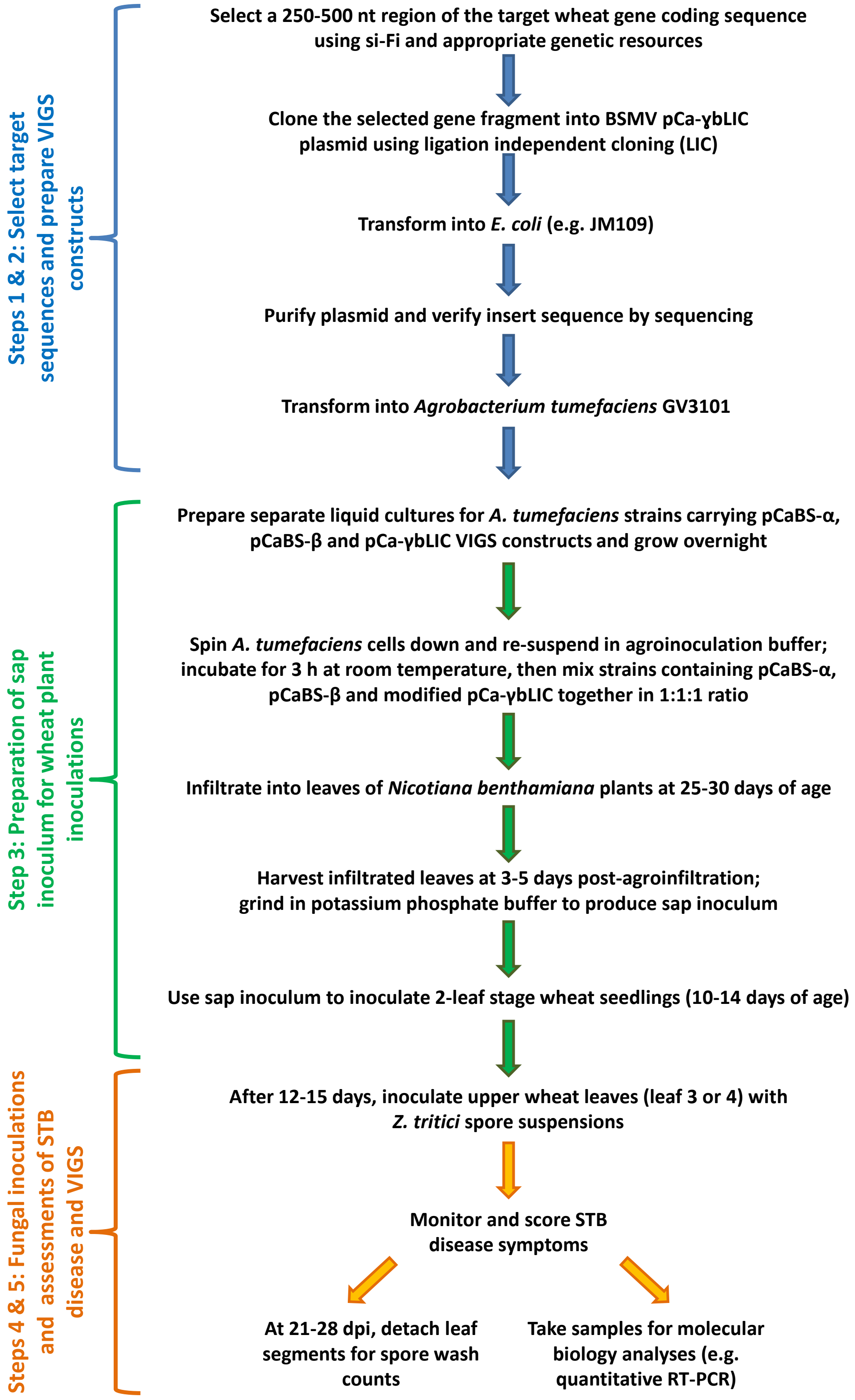

Supplement: Supplementary Fig. 1 [file mmc1.pdf]
